# Effectiveness of neutral honey as a tissue fixative in histopathology

**DOI:** 10.12688/f1000research.122598.2

**Published:** 2023-07-21

**Authors:** Nasar Alwahaibi, Buthaina Al Dhahli, Halima Al Issaei, Loai Al Wahaibi, Shadia Al Sinawi

**Affiliations:** 1 Allied Health Sciences Department, College of Medicine and Health Sciences, Sultan Qaboos University, Muscat, 123, Oman; 2 Pathology Department, Sultan Qaboos University Hospital, Sultan Qaboos University, Muscat, 123, Oman

**Keywords:** Histopathology, fixation, honey, formalin, neutral buffered

## Abstract

**Background:** In routine histopathology, 10% neutral buffered formalin (NBF) is the choice fixative. However, formalin is a human carcinogen, so there is a necessity for a safer alternative. To the best of our knowledge, neutral honey, not natural or artificial honey, has not been tested to fix histological samples. This study determined the effectiveness of neutral buffered honey and other types of fixatives to fix histological tissues.

**Methods:** The study was conducted between July 2019 and August 2020 at Sultan Qaboos University, Oman. Sections from three rat livers, kidneys, and stomach tissues were fixed with 10% NBF, neutral buffered Sumer honey, neutral buffered date honey, formalin, Sumer honey, date honey, alcoholic formalin, alcoholic Sumer honey, and alcoholic date honey for 24 hours. Samples were stained with hematoxylin and eosin (H&E), special stains, and vimentin methods. Three expert biomedical scientists then evaluated the fixed and stained samples for the quality of all sections. The fixation ability of the different honey solutions was then compared to 10% NBF and the utility was determined using nuclear and cytoplasmic criteria, specificity, and intensity.

**Results:** H&E showed adequate staining in all groups compared to 10% NBF. The specificity and intensity of all groups for the Periodic acid–Schiff method were identical to 10% NBF except for Sumer honey and alcoholic date honey. Vimentin showed comparable findings with 10% NBF as there were no significant differences.

**Conclusions:** The findings of this study encourage the use of honey, including neutral, as a possible safe substitute fixative for formalin, however, further experiments on larger specimens should be conducted.

## Introduction

Fixation is an initial and critical tissue processing step for microscopy in histopathology. ^1^ Fixation preserves the tissues' life-like condition by preventing autolysis and bacterial putrefaction. ^2^ 10% neutral buffered formalin (NBF) is the preferred fixative of choice because it is readily available and internationally accepted. Its preparation is easy, fast, cheap, and has long-term storability. ^3^^,^^4^ However, the International Agency for Research on Cancer (IARC) and US Environmental Protection Agency (EPA) classified formaldehyde, which is the primary component of formalin, as a human carcinogen. ^5^

Therefore, safer alternative fixatives should replace formalin, such as natural fixatives that are eco-friendly, economical, and readily available substances. Examples of natural fixatives are honey, sugar, jaggery, molasses, saline, rose water, and coconut oil. Honey Bee is a mixture of sugars, minerals, trace elements, vitamins such as vitamin C, and antioxidants such as pinobanksin, pinocembrin, hydrogen peroxide, chrysin, and catalase. ^6^ Combined, these compounds give honey its anti-autolytic, antimicrobial, antiviral, antimutagenic, and antioxidant effects. These honey features are known for several centuries. ^7^ Bee honey is acidic and also possesses preserving, dehydrating, and tissue hardening properties. ^3^ In addition, it can penetrate the deepest tissue. ^3^ These properties make the honey a potential tissue fixative. However, tissues fixed in honey at low pH are less rigid after fixation and have homogenized connective tissue, a breach in the continuity of sections, folding of the tissue sections, and growth of molds over some time. ^1^^,^^3^^,^^8^^,^^9^

The present study was designed to find a safe substitute fixative for formalin without compromising the quality staining criteria of the tissue sections. The hypothesis was that neutral honey fixative or other experimented honey fixed rat tissues better or similar to that of 10% NBF. Neutral honey is honey where the pH is around seven whereas natural honey usually has a low pH. It is recommended that the pH fixatives be kept near seven in order to achieve optimal results. ^10^ We thought that increasing the pH of the honey might overcome the disadvantages associated with low pH honey fixatives. To the best of our knowledge, neutral honey has not been experimented with to fix histological tissues. Thus, we aimed to examine the effectiveness of neutral buffered honey and other types of honey fixatives to fix histological tissues.

## Methods

### Ethical considerations

The study was ethically approved by the Ethics Committee for Animal Use in Research, College of Medicine and Health Sciences, Sultan Qaboos University, Oman (Ethical Clearance number SQU/EC-AUR/2021-2022-15). Three Wister rats (200 g) were retained for two days in a room measured setting (a temperature of 20 ± 2°C, relative humidity of about 60%, with a 12-hour light-dark cycle). They were provided *ad libitum* with an additive-free standard diet (Oman Flour Mills, Muscat, Oman) and tap water. The rats were euthanized with an overdose of ketamine (75 mg/kg) and xylazine (5 mg/kg) *via* intraperitoneal injection. All efforts were made to ameliorate any suffering of animals as all procedures were carried out per the national, and international laws and policies. In addition, this study is reported in line with The Animal Research: Reporting on in vivo Experiments (ARRIVE) guidelines. ^11^^,^^27^ The cost of formalin, Sumer honey, and date honey were obtained from local suppliers.

### Study design

Two common natural honeys were used as fixatives, namely Sumer and date. Sumer is the most well-known and one of the finest types of Omani natural honey. It is light black in color and produced from Omani red dwarf bees (Apis florea). It is very expensive honey as it is produced in small amounts and usually found in the caves of the mountains, therefore, it is difficult to acquire. Date honey is very common in Oman as it is extracted from dates. It is slightly brown in color and not expensive. In this study, we evaluated nine groups of fixatives, including neutral buffered Sumer honey, neutral buffered date honey, formalin, Sumer honey, date honey, alcoholic formalin, alcoholic Sumer honey, and alcoholic date honey. The pH for these fixatives were 7.20, 7.25, 7.27, 3.62, 3.56, 5.15, 3.95, 5.0, and 5.85, respectively. These groups were compared with the gold standard fixative, which is 10% NBF. The neutral buffer was made with sodium phosphate monobasic (0.4 g), sodium phosphate dibasic (0.65 g), formalin (10 ml), and distilled water (90 ml). 10% fixatives contain 10 ml (Sumer and date honeys and formalin) and 90 ml of distilled water. 10% alcoholic fixatives contain 10 ml (Sumer and date honeys and formalin) and 90 ml ethanol. In this study, we have chosen experimental conditions such as a temperature of 18–23°C, 24 hours fixation time, tissue thickness of 3 mm, and fixation volume of 1:10. In fact, 1:10 fixation volume has been reported by many researchers and found to be the most recommended ratio. ^12^^,^^13^ In addition, during our pilot study we used 5, 10, and 15% honey fixative concentrations for 24 and 48 hours fixation time and found that 10% for 24 hours fixation preserved tissue morphology well.

### Specimen processing

Nine samples were fixed in each group: three uniform-thickness samples (3 mm) from each organ (liver, kidney, and stomach) were obtained from the rats. In order to have a uniform thickness for all groups, tissues were cut using the Cutmate forceps (Forceps for 2/3/4 mm tissue blocks, Code No 62359, Milestone s.r.l, Sorisole, Italy). The tissues were immediately placed in fixatives for 24 hours at room temperature (18–23°C). After fixation, all tissues were given the same treatment. As previously described, all tissues were processed using an automated histoprocessor (Spin Tissue Processor Microm STP 120; Thermo Scientific, Walldorf, Germany). ^14^ Processing included dehydration in ethanol, clearing in xylene, and infiltration in paraffin wax. Afterward, 3 μm sections were cut using a rotatory microtome (Leica RM2135, Nussloch, Germany). One slide was obtained from each organ ( *i.e.*, three from the liver, three from the kidney, and three from the stomach). Thus, for all three organs, nine slides were obtained and for all nine groups, 81 slides were obtained. Differences in microtomy were observed.

**Hematoxylin and eosin (H&E) and special stains**

All 81 slides were stained with H&E. ^14^ In addition, a number of special stains were used to stain slides in different groups. Jones' Methenamine silver stain (JMS) to stain glomerular basement membranes in the kidney slides. Gordon and Sweets method was used to stain reticular fibers in the liver slides. The periodic acid–Schiff (PAS) method was used to detect basement membranes of glomerular capillary loops and tubular epithelium. ^14^ All special stains were performed using the Ventana BenchMark Special Stains system (Ventana Medical Systems, Inc., Code No: 06657389001, Tucson, AZ, USA). Positive controls (human kidney for JMS and PAS, and human liver for G&S; Pathology Department, Sultan Qaboos University Hospital) were run simultaneously.

**Immunohistochemistry**

Three additional kidney tissue samples cut at 4 μm using a rotatory microtome (Leica RM2135; Nussloch, Germany) were obtained for vimentin staining. Slides were deparaffinized, rehydrated and epitope retrieved by pre-treatment (PT) link (Code PT200, Agilent Dako, CA, USA). All slides were then washed three times in phosphate buffered saline (PBS) each for a min. Following which, slides were incubated with a polyclonal primary antibody against vimentin (host species: rabbit; Abcam Cat# ab137321, RRID:AB_2921312) at 1:1000 dilution for 30 minutes. Slides were then washed with PBS three times each for 5 min followed by incubation for 30 min with a secondary antibody (Envision Flex, High pH (Link), Hrp. Rabbit/Mouse; Agilent Cat# K8000, RRID:AB_2890017) and then washed with PBS three times each for 5 min. After that, the reaction was visualized using 3,3′-Diaminobenzidine (Code No K3468, Dako, CA, USA) for 2 min. Next, slides were counterstained with Mayer's hematoxylin for 2 min and then washed for 2 min in running tap water. Finally, slides were dehydrated, cleared, and mounted in dibutyl phthalate polystyrene xylene. Placental tissue previously known to be positive was used as a positive control for vimentin (Pathology Department, Sultan Qaboos University Hospital).

### Evaluation and data analysis

All slides were examined by light microscopy (BX40, Olympus Optical Co, Tokyo, Japan) attached to a camera (DP71 controller, Olympus Optical Co, Tokyo, Japan). For H&E evaluation ( Table 1), if the score was ≤ 2, graded as inadequate, and if the score was 3-5, graded as adequate. ^15^^,^^16^ For special stains, specificity, and intensity, were used and graded either negative (0), weak (1), moderate (2), or strong (3). For special stains, specificity, and intensity, were used and graded either negative (0), weak (1), moderate (2), or strong (3). For immunohistochemistry, the intensity was used and graded either negative (0), weak (1), moderate (2), or strong (3), and for the background is the inverse. Three senior biomedical scientists working in a histopathology laboratory blindly evaluated all the slides, such that all slides had three results for each parameter. Then the average score of each parameter was obtained. The data were analyzed using IBM SPSS Statistics (RRID:SCR_016479) software version 23 (IBM Corp., Armonk, New York, United States). For Fisher's exact test: we compared two staining criteria, for example, adequate and inadequate for nuclear staining. For the ANOVA test, we compared more than two criteria: the grade (negative, weak, moderate, and strong) of specificity for each fixative group. The comparison was with the NBF as the gold standard in all fixative groups. A P-value of less than 0.05 was considered statistically significant.

**Table 1.** Histomorphological criteria using hematoxylin and eosin staining method.

Parameters	Score & criteria	
Nuclear staining	Acceptable = 1 Round, smooth and clear nuclear membrane	Unacceptable = 0 Granular, disintegrated, and out of focus
Cytoplasmic staining	Acceptable = 1 Intact cytoplasmic membrane and transparent cytoplasm	Unacceptable = 0 Disintegrated cytoplasmic membrane, granular cytoplasm, and out of focus
Tissue morphology	Preserved = 1 Absence of folds, no overlap, and maintained N: C ratio	Unpreserved = 0 Overlapping cells, folded, and disintegrated cells
Clarity of staining	Present = 1 The crispness in staining and transparency	Absent = 0 Obliterates the nucleus and cytoplasm
Uniformity of staining	Present = 1 Uniformly stained throughout the individual cell	Absent = 0 Stained in different shades of color in an individual cell

## Results

**Figure 1.**
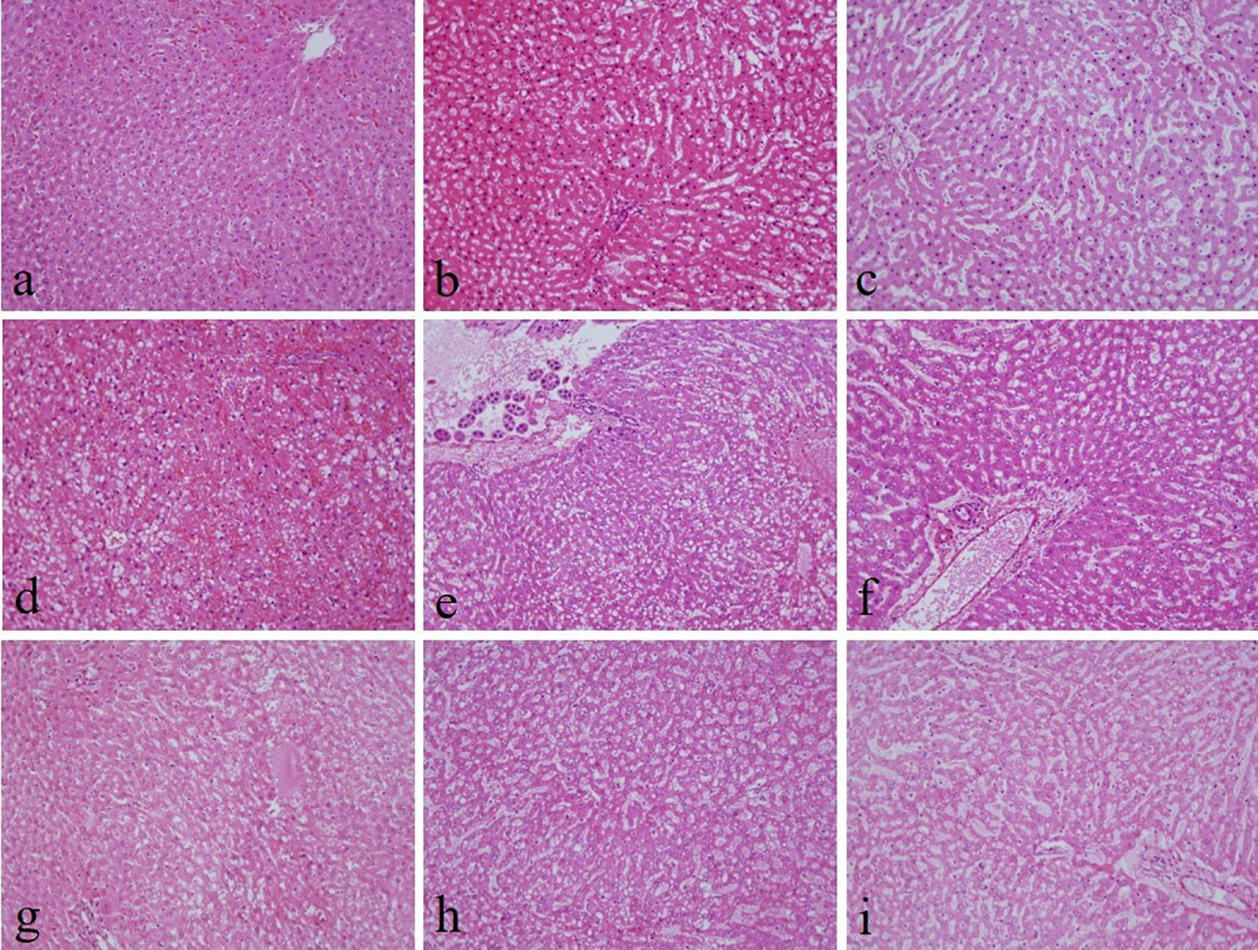
Hematoxylin and eosin-stained liver sections of different fixative groups. (a) 10% neutral buffered formalin, (b) 10% neutral buffered Sumer honey, (c) 10% neutral buffered date honey, (d) 10% formalin, (e) 10% Sumer honey, (f) 10% date honey, (g) 10% alcoholic formalin, (h) 10% alcoholic Sumer honey and (i) 10% alcoholic date honey (magnification, ×200).

There were no noticeable differences in sectioning, formation of ribbons, and floating on the water bath in all groups. There was no significant difference in the nuclear staining and uniformity of staining among all the groups ( Table 2). ^27^ By contrast, 10% neutral buffered Sumer honey, 10% neutral buffered date honey, 10% Sumer honey, and 10% date honey fixed tissues had significantly inferior cytoplasmic staining compared to 10% NBF ( Figure 1).

**Table 2.** Comparison of different honey fixative groups on rat liver, kidney, and stomach using hematoxylin and eosin staining method.

Groups	10% NB			10% fixatives			10% alcoholic fixatives		
	NBF	NBS	NBD	F	S	D	AF	AS	AD
Nuclear staining Adequate	9	6	6	9	8	8	9	9	9
Inadequate	0	3	3	0	1	1	0	0	0
P-value		0.21	0.21	1.00	1.00	1.00	1.00	1.00	1.00
Cytoplasm staining Adequate	9	0	3	9	3	3	9	9	6
Inadequate	0	9	6	0	6	6	0	0	3
P-value		<0.001	0.01	1.00	0.01	0.01	1.00	1.00	0.21
Cell morphology preserved	9	5	6	9	8	3	9	3	0
Unpreserved	0	4	3	0	1	6	0	6	9
P-value		0.08	0.21	1.00	1.00	0.01	1.00	0.01	<0.001
Clarity of staining Present	9	0	3	9	6	9	9	6	6
Not present	0	9	6	0	3	0	0	3	3
P-value		<0.001	0.01	1.00	0.21	1.00	1.00	0.21	0.21
Uniformity of staining Present	9	6	6	9	9	6	9	9	9
Not present	0	3	3	0	0	3	0	0	0
P-value		0.21	0.21	1.00	1.00	0.21	1.00	1.00	1.00

NBF: neutral buffered formalin; NBS: neutral buffered Sumer honey; NBD: neutral buffered Date honey; F: formalin; S: Sumer honey; D: Date honey; AF: Alcoholic formalin; AS: Alcoholic Sumer honey; AD: Alcoholic Date honey. Statistical significance Fisher’s exact test.

**Figure 2.**
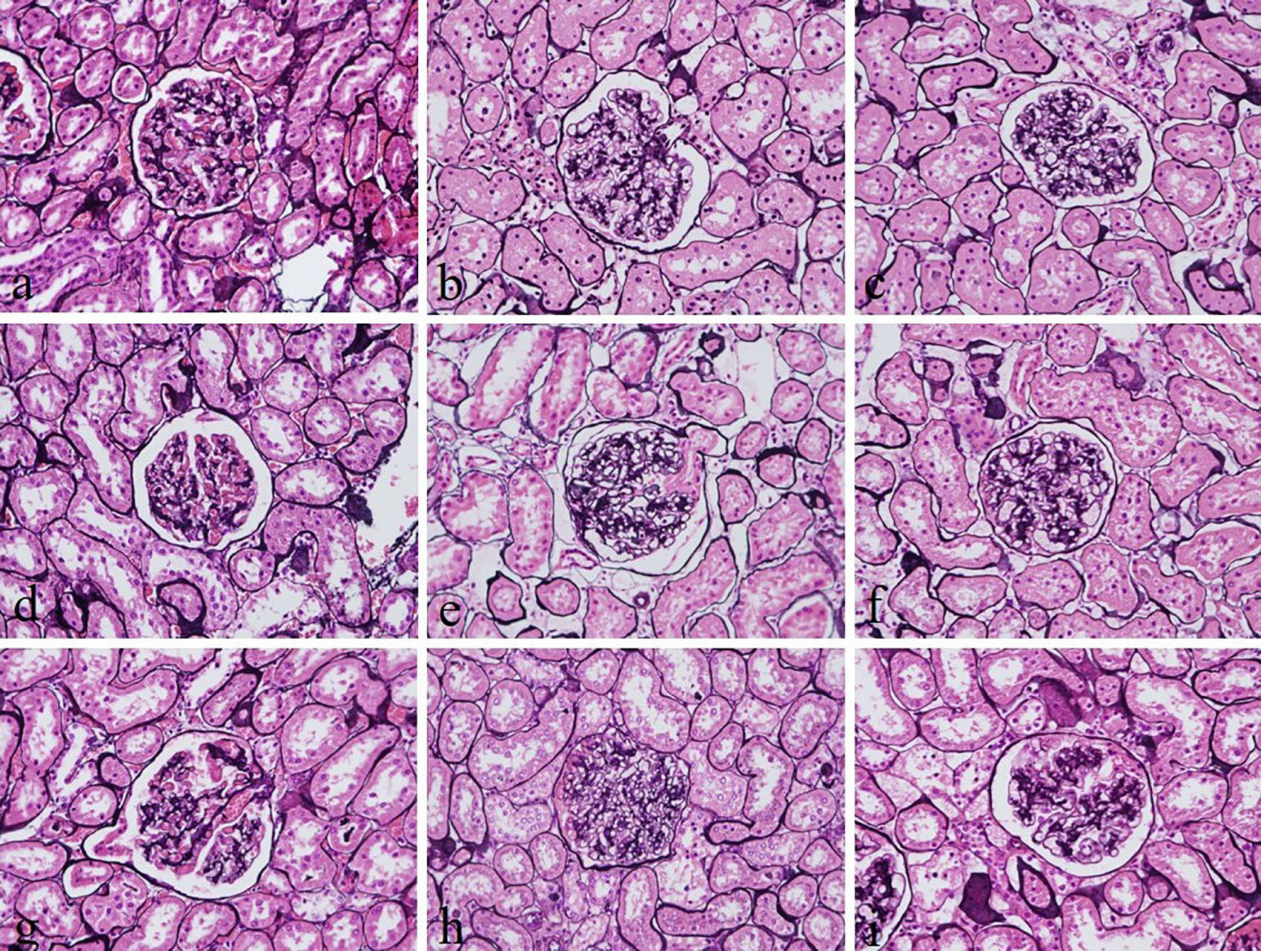
Jones’ Methenamine silver-stained kidney sections of different fixative groups. (a) 10% neutral buffered formalin, (b) 10% neutral buffered Sumer honey, (c) 10% neutral buffered date honey, (d) 10% formalin, (e) 10% Sumer honey, (f) 10% date honey, (g) 10% alcoholic formalin, (h) 10% alcoholic Sumer honey and (i) 10% alcoholic date honey (magnification, ×400).

**Figure 3.**
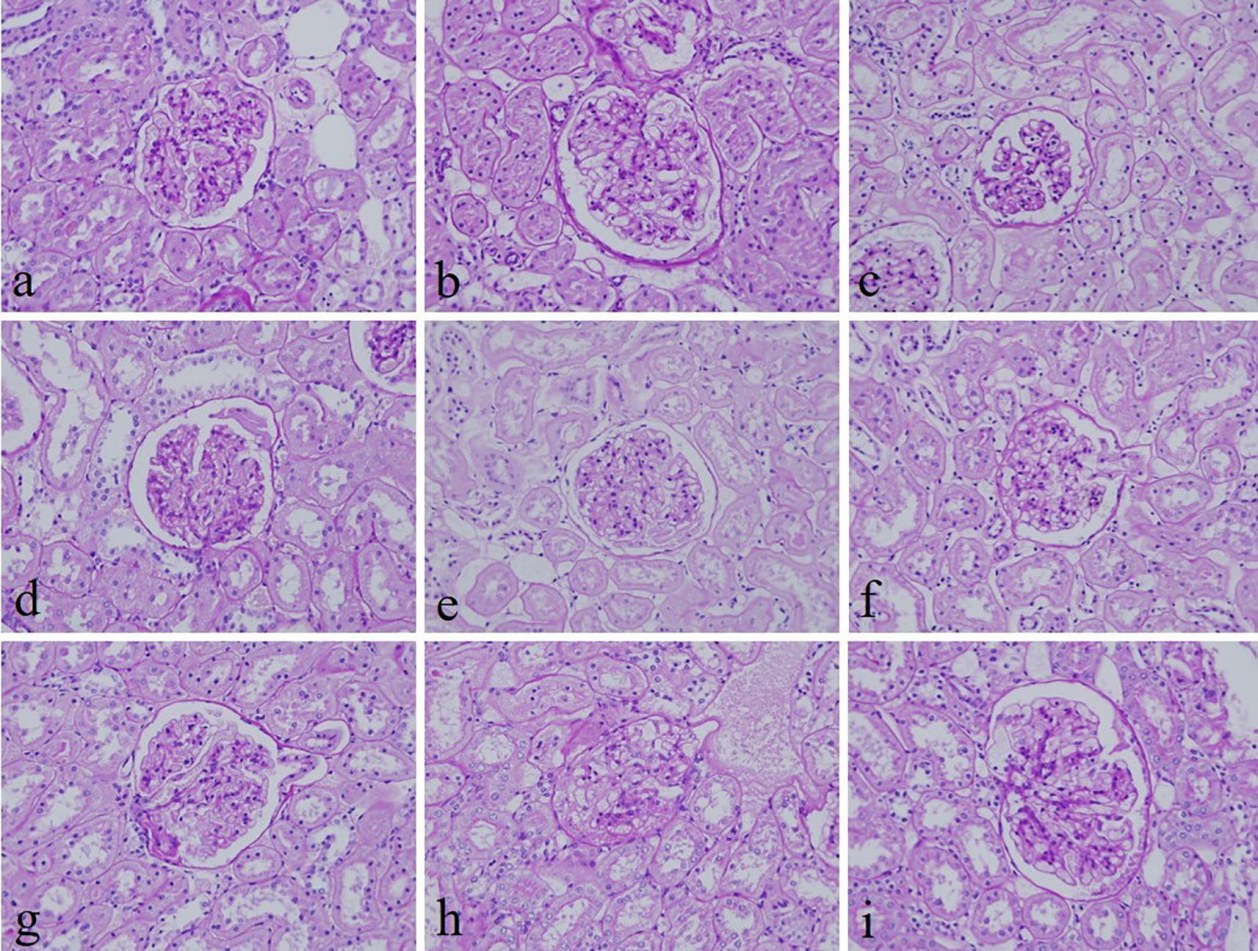
Periodic acid–Schiff-stained kidney sections of different fixative groups. (a) 10% neutral buffered formalin, (b) 10% neutral buffered Sumer honey, (c) 10% neutral buffered date honey, (d) 10% formalin, (e) 10% Sumer honey, (f) 10% date honey, (g) 10% alcoholic formalin, (h) 10% alcoholic Sumer honey and (i) 10% alcoholic date honey (magnification, ×400).

**Figure 4.**
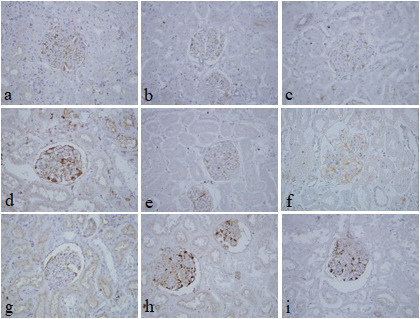
Vimentin-stained kidney sections of different fixative groups. (a) 10% neutral buffered formalin, (b) 10% neutral buffered Sumer honey, (c) 10% neutral buffered date honey, (d) 10% formalin, (e) 10% Sumer honey, (f) 10% date honey, (g) 10% alcoholic formalin, (h) 10% alcoholic Sumer honey and (i) 10% alcoholic date honey (magnification, ×400).

The intensity and specificity of JMS in 10% Sumer and date honeys and 10% alcoholic Sumer honey were similar to that of 10% NBF ( Figure 2). In addition, the specificity and intensity of all groups for PAS were comparable with 10% NBF. However, the intensity of PAS in 10% Sumer honey and 10% alcoholic date honey were inferior in comparison with 10% NBF ( Figure 3). All honey groups showed weak staining of the reticulin fibers using the Gordon and Sweets method ( Table 3). Immunohistochemical staining with vimentin showed comparable findings with 10% NBF as there were no significant differences noted in intensity and background for all groups ( Figure 4). In addition, no background staining was observed in all groups.

**Table 3.** Comparison of different fixative groups on rat liver and kidney using three special stains and vimentin.

Tissues	Specificity/intensity	Groups	10% NB			10% fixatives			10% alcoholic fixatives		
			NBF	NBS	NBD	F	S	D	AF	AS	AD
G&S Liver	Specificity	Negative	0	1	1	0	1	3	0	1	1
		Weak	0	2	2	0	2	0	0	2	2
		Moderate	1	0	0	1	0	0	1	0	0
		Strong	2	0	0	2	0	0	2	0	0
		P-value		<0.001	<0.001	1.000	<0.001	<0.001	1.000	<0.001	<0.001
	Intensity	Negative	0	1	2	0	1	3	0	2	2
		Weak	0	2	1	0	2	0	0	1	1
		Moderate	2	0	0	2	0	0	2	0	0
		Strong	1	0	0	1	0	0	1	0	0
		P **-**value		<0.001	<0.001	1.000	<0.001	<0.001	1.000	<0.001	<0.001
JMS Kidney	Specificity	Negative	0	0	0	0	1	0	0	0	0
		Weak	0	1	1	0	0	2	0	1	1
		Moderate	2	2	2	0	1	1	3	1	2
		Strong	1	0	0	3	1	0	0	1	0
		P-value		0.99	0.99	1.000	0.99	0.72	1.000	0.99	0.99
	Intensity	Negative	0	0	0	0	0	0	0	0	0
		Weak	0	1	3	0	0	1	0	0	3
		Moderate	0	2	0	0	2	2	3	2	0
		Strong	3	0	0	3	1	0	0	1	0
		P-value		0.84	0.01	1.000	1.000	0.84	1.000	1.000	0.01
PAS Kidney	Specificity	Negative	0	0	0	0	0	0	1	0	0
		Weak	0	1	1	0	0	0	0	0	0
		Moderate	0	2	0	0	1	3	1	0	2
		Strong	3	0	2	3	2	0	1	3	1
		P-value		0.94	0.94	1.000	1.000	1.000	0.94	1.000	1.000
	Intensity	Negative	0	0	0	0	0	0	1	0	0
		Weak	0	0	0	0	3	2	1	0	3
		Moderate	1	3	3	0	0	1	1	0	0
		Strong	2	0	0	3	0	0	0	3	0
		P-value		1.000	1.000	1.000	0.01	0.13	0.13	1.000	0.01
Vimentin Kidney	Intensity	Negative	0	0	0	0	0	0	0	0	0
		Weak	1	3	3	0	2	3	2	0	1
		Moderate	2	0	0	2	1	0	1	2	1
		Strong	0	0	0	1	0	0	0	1	1
		P-value		0.49	0.49	0.97	0.97	0.49	0.97	0.97	1.000
	BG	Negative	2	2	1	1	0	3	1	0	2
		Weak	1	1	2	1	1	0	2	1	1
		Moderate	0	0	0	1	2	0	0	1	0
		Strong	0	0	0	0	0	0	0	1	0
		P-value		1.000	1.000	0.94	0.32	1.000	1.000	0.32	1.000

BG: Background; NBF: Neutral buffered formalin; NBS: Neutral buffered Sumer honey; NBD: Neutral buffered Date honey; F: Formalin; S: Sumer honey; D: Date honey; AF: Alcoholic formalin; AS: Alcoholic Sumer honey; AD: Alcoholic Date honey; G&S: Gordon and Sweets; JMS: Jones' Methenamine silver stain; PAS: Periodic acid–Schiff. Statistical significance by ANOVA test.

Sumer honey is more expensive than formalin. One liter of Sumer costs 60 Omani Rial (OMR), equivalent to 155.79 USD, whereas one liter formalin costs OMR 1.6, equivalent to USD 4.15. One liter of date honey costs USD 5.19 (OMR 2.00).

## Discussion

The aim of the present study was to find a safe substitute fixative for formalin, which is a human carcinogen. To the best of our knowledge, this is the first study to evaluate honey as neutral buffered honey similar to that of the NBF. The honeys used in this study had overall similar findings to that of NBF. This study presents two types of honey: Sumer (produced by red dwarf bees) and Date honey (produced from dates). These two types of honey are common in Oman. Sumer honey is also available in Saudi Arabia, United Arab Emirates, India, Iran, Iraq, Jordan, Yemen, Thailand, Cambodia, Vietnam, China, and Sudan. Date honey is found in any country that produces dates such as Libya, Algeria, Iran, Iraq, Jordan, Yemen, Saudi Arabia, and United Arab Emirates. Other countries might have different types of honey.

The present study evaluates three important aspects of honey fixation: H&E, special stain, and immunohistochemistry. In both types of honeys and in all groups, H&E results showed that the overall quality of tissue staining was comparable with 10% NBF. The nucleus was well preserved with precise nuclear details. It is known that honey bee contains ascorbic acid and various vitamins, carbohydrates, minerals, and trace elements. Thus, it gives honey a low pH. Low pH fixative is suitable for nuclear staining. ^17^

The findings of the present study are in line with other studies that have reported that 10% honey bee, using rat liver and kidney tissues at room temperature for 24-hour fixation, gave comparable results with those obtained by formalin-fixed control tissues. ^18^ Another similar study that used the same staining criteria to evaluate honey bee as a substitute for formalin found that 10% honey is as good as 10% NBF and suggested that honey is a safe alternative for formalin. ^6^ Recently, two studies in cytology showed that 20% honey bee fixed oral smears had acceptable nuclear and cytoplasmic staining, well-preserved cell morphology, clarity, and uniformity of staining comparable to ethanol, which is the gold standard fixative in cytology, with no statistical difference between both fixatives. ^19^^,^^20^ However, in the current study, cytoplasmic staining was inadequate in neutral buffered Sumer honey, neutral buffered date honey, 10% Sumer honey, and 10% date honey. We thought by increasing the pH in neutral buffered Sumer honey and neutral buffered date honey fixatives would enhance cytoplasmic staining. The results of the present study are in concordance with another similar study where they compared formalin fixed tissues with honey bee fixed tissues and concluded that the nuclear details are well established compared to cytoplasmic details. ^9^

Reticular fibers in all groups were not well demonstrated. The staining was weak. This finding disagrees with another study, which reported that reticular fibers using silver impregnation were well demonstrated using 10% honey bee as a fixative. ^20^ However, the demonstration of glomerular basement membranes in the kidney by JMS in 10% Sumer and date honey and 10% alcoholic Sumer honey were similar to 10% NBF fixed sections. Srii *et al.*, evaluated the efficacy of 10% honey bee as a fixative agent to preserve cellular and structural characteristics using different special stains. They found that Masson's trichrome and Van Gieson staining results are similar to those fixed by formalin. ^21^ Similarly, the specificity and staining intensity of PAS on honey fixed tissues were comparable with 10% NBF. ^4^ Our results align with this study where PAS stain in all groups revealed similar findings with 10% NBF. However, the intensity for only 10% Sumer honey and 10% alcoholic date honey was inferior compared to 10% NBF.

In this study, both types of honey in H&E and special stains showed the absence of red blood cells in the liver, kidney, and stomach tissues. When red blood cells are exposed to hydrogen peroxide, which is a component of honey, this would make cellular changes that lead to alterations in phospholipid organization and cell shape, and membrane deformability. Hydrogen peroxide has the ability to form a covalent complex with hemoglobin and spectrin, which are specific structures of red blood cells. ^22^ This may explain why honey masks the staining of red blood cells.

In the current study, vimentin as an immunohistochemical marker was evaluated. In routine immunohistochemistry, this marker is used as an internal control for detecting cytoplasmic staining. ^23^^,^^24^ Vimentin in all pine honey groups showed similar findings of 10% NBF as similarly reported by Özkan *et al.*, in which honey fixed ki-67 and vimentin markers in different fresh tissues, including endometrium, breast, placenta, uterus, omentum, suprarenal, stomach, and lung similar to NBF. ^2^ Gunter and Bryant reported good staining levels without antigen retrieval for common leukocyte antigen, cytokeratin AE1/AE3, and epithelial membrane antigen in breast tumor samples treated with pure honey. ^25^ In addition, the demonstration of vimentin and pan-cytokeratin in gingiva tissues using 10% honey was similar to formalin-fixed tissues. ^25^ Similarly, the immunohistochemical demonstration of pan-cytokeratin was comparable to using 20% pure honey fixative in fresh goat oral mucosa. ^26^

In comparison with formalin, the cost of Sumer honey is very expensive, however, date honey costs are almost similar to formalin. Thus, the cost-benefit balance between the safety of laboratory workers in histopathology and good quality staining should be considered.

Several limitations of our study are worth noting. First, we should point out that large tissues or small biopsies were not evaluated; differently sized tissues would produce more meaningful results. Second, only one immunohistochemical tumor marker was assessed; a wide range of immunohistochemical markers would improve our knowledge on how useful honey is as a potential fixative. Third, a limited number of special stains was evaluated. Fourth, the sample number was small. Finally, DNA/RNA extraction from honey-fixed specimens was not assessed.

## Conclusions

Neutral honey is potentially a safer substitute fixative for formalin, however, further experiments on larger and different specimens and additional special stains and immunohistochemical markers should be conducted.

## Data availability

### Underlying data

Zenodo: Effectiveness of neutral honey as a tissue fixative in histopathology. https://doi.org/10.5281/zenodo.6591173. ^27^

This project contains the following underlying data:

- Author_Checklist_-_Full.pdf (ARRIVE checklist)
- H&E slides evaluation.xlsx (81 H&E slides evaluation)
- Figure 5. jpg (Gordon and Sweets stained-liver sections of different fixative groups: (a) 10% neutral buffered formalin, (b) 10% neutral buffered Sumer honey, (c) 10% neutral buffered Date honey, (d) 10% formalin, (e) 10% Sumer honey, (f) 10% Date honey, (g) 10% alcoholic formalin, (h) 10% alcoholic Sumer honey and (i) 10% alcoholic Date honey (x400))
- Figure 6. jpg (Hematoxylin and eosin-stained stomach sections of 10% neutral buffered formalin (magnification, x200))
- Figure 7. jpg (Hematoxylin and eosin-stained stomach sections of 10% neutral buffered Sumer honey (magnification, x200))
- Figure 8. jpg (Hematoxylin and eosin-stained stomach sections of 10% neutral buffered date honey (magnification, x200))
- Figure 9. jpg (Hematoxylin and eosin-stained stomach sections of 10% formalin (magnification, x200)).
- Figure 10. jpg (Hematoxylin and eosin-stained stomach sections of 10% Sumer honey (magnification, x200))
- Figure 11. jpg (Hematoxylin and eosin-stained stomach sections of 10% date honey (magnification, x200))
- Figure 12. jpg (Hematoxylin and eosin-stained stomach sections of 10% alcoholic formalin (magnification, x200))
- Figure 13. jpg (Hematoxylin and eosin-stained stomach sections of 10% alcoholic Sumer honey (magnification, x200))
- Figure 14. jpg (Hematoxylin and eosin-stained stomach sections of 10% alcoholic date honey (magnification, x200))

### Reporting guidelines

Zenodo: ARRIVE checklist for ‘Effectiveness of neutral honey as a tissue fixative in histopathology’. https://doi.org/10.5281/zenodo.6591173. ^27^

Data are available under the terms of the Creative Commons Attribution 4.0 International license (CC-BY 4.0).
