# Supplementary material for: Effectiveness of neutral honey as a tissue fixative in histopathology
Source: F1000Res. 2023 Nov 9;11:1014. Originally published 2022 Sep 7. [Version 3] doi: 10.12688/f1000research.122598.3 (PMC10905026; doi:10.12688/f1000research.122598.3)
Supplement: Peer review history

# Reviewer report: Reviewer response for version 3

**DOI:** 10.5256/f1000research.158419.r220717

**Published:** 2024-02-27

**Authors:** Phyu Synn Oo

**Affiliations:** 1 International Medical University, Kuala Lumpur, Malaysia

This is an interesting study with promising results.

 There are a few fragmented sentences and grammatical errors that the authors need to revise. 

1. Pictures in Figure 4 are very low quality compared to other figures. It's better to change with high-quality pictures. 
2. Abstract - instead of vimentin methods, I suggest using vimentin staining with IHC or IHC (vimentin). 
3. Pg. 3 - Methods - fragmented sentences should be revised. 
4. Pg. 4 - Specimen processing and Pg. 5 - results - In specimen processing, it was mentioned that there were differences in microtomy, but results said no noticeable differences in sectioning, formation of ribbons, and floating on the water bath. The authors need to revise it. 
5. Pg. 4 - Evaluation and data analysis - duplication of the sentences and sentence fragments. 
6. Fonts in text and figures/tables legends are different. I suggest the authors follow a consistent format. 
7. Pg. 8 - Discussion - "Pine honey" is used for the first time. Clarify which honey do you prefer? 

Is the work clearly and accurately presented and does it cite the current literature?

Yes

If applicable, is the statistical analysis and its interpretation appropriate?

I cannot comment. A qualified statistician is required.

Are all the source data underlying the results available to ensure full reproducibility?

Yes

Is the study design appropriate and is the work technically sound?

Yes

Are the conclusions drawn adequately supported by the results?

Partly

Are sufficient details of methods and analysis provided to allow replication by others?

Yes

Reviewer Expertise:

Pathology, Molecular Biology

I confirm that I have read this submission and believe that I have an appropriate level of expertise to confirm that it is of an acceptable scientific standard.

# Reviewer report: Reviewer response for version 2

**DOI:** 10.5256/f1000research.153237.r189341

**Published:** 2023-11-02

**Authors:** Marie-Odile Benoit-Biancamano

**Affiliations:** 1 Universite de Montreal, Montreal, Québec, Canada

Figure 4 seems to have been submitted in low resolution and appears blurry.

**Minor comments**

 Abstract (conclusions): The findings of this study encourage the use of honey, including neutral **buffered honey**, as a possible safe substitute fixative for formalin, however, further experiments on larger specimens should be conducted.

Is the work clearly and accurately presented and does it cite the current literature?

Yes

If applicable, is the statistical analysis and its interpretation appropriate?

Partly

Are all the source data underlying the results available to ensure full reproducibility?

Yes

Is the study design appropriate and is the work technically sound?

Yes

Are the conclusions drawn adequately supported by the results?

Partly

Are sufficient details of methods and analysis provided to allow replication by others?

Yes

Reviewer Expertise:

Veterinary pathology

I confirm that I have read this submission and believe that I have an appropriate level of expertise to confirm that it is of an acceptable scientific standard.

# Response

**Published:** 2023-11-05

**Authors:** Nasar Alwahaibi

**Affiliations:** Biomedical Science, Sultan Qaboos University, Muscat, Oman

We would like to thank the reviewer further for the comments.

 Figure 4 seems to have been submitted in low resolution and appears blurry.

**Response**

**Figure 4 has been replaced with a better resolution.**

 Minor comments

 Abstract (conclusions): The findings of this study encourage the use of honey, including neutral buffered honey, as a possible safe substitute fixative for formalin, however, further experiments on larger specimens should be conducted.

**Response**

**As suggested, the conclusion has been modified.**

# Reviewer report: Reviewer response for version 2

**DOI:** 10.5256/f1000research.153237.r211041

**Published:** 2023-10-26

**Authors:** Samah Kandeel

**Affiliations:** 1 Histology & Cell Biology, Tanta University, Tanta, Egypt

Dear editor:

 Effectiveness of neutral honey as a tissue fixative in histopathology is a very attractive topic to avoid the carcinogenic effect of formalin as it is already the commonly used fixative at histology Lab.

 However, some issues are raised; one of them is that the way that we can obtain Sumer honey as author mentioned it is very costly and difficult to be obtained and any applicable materials should be easily obtained.

 Also, the preferred pH for tissue fixation should be between 6-8; while at the present research authors used pH of the prepared fixatives "The pH for these fixatives were 7.20, 7.25, 7.27, 3.62, 3.56, 5.15, 3.95, 5.0, and 5.85, respectively." so, I think pH monitoring should be considered.

 Figure 1: needed to be at higher magnification to be show the tissue details clearly.

 Figure 4: negative immunohistochemical image is needed; also testing another immunohistochemical marker should be applied as vimentin reaction is not so obvious at this figure.

Is the work clearly and accurately presented and does it cite the current literature?

Yes

If applicable, is the statistical analysis and its interpretation appropriate?

Partly

Are all the source data underlying the results available to ensure full reproducibility?

Partly

Is the study design appropriate and is the work technically sound?

Partly

Are the conclusions drawn adequately supported by the results?

Yes

Are sufficient details of methods and analysis provided to allow replication by others?

Yes

Reviewer Expertise:

Histology and cell biology, Immunohistochemistry, cell immunology

I confirm that I have read this submission and believe that I have an appropriate level of expertise to confirm that it is of an acceptable scientific standard, however I have significant reservations, as outlined above.

# Response

**Published:** 2023-11-01

**Authors:** Nasar Alwahaibi

**Affiliations:** Biomedical Science, Sultan Qaboos University, Muscat, Oman

**We would like to take this opportunity to express our thanks to the reviewer for the positive feedback and helpful comments.**

**Below are our responses, point-by-point to the queries of the reviewer.**

 Effectiveness of neutral honey as a tissue fixative in histopathology is a very attractive topic to avoid the carcinogenic effect of formalin as it is already the commonly used fixative at histology Lab.

 However, some issues are raised; one of them is that the way that we can obtain Sumer honey as author mentioned it is very costly and difficult to be obtained and any applicable materials should be easily obtained.

**Response**

**Thank you. Unfortunately, Sumer honey is expensive as usually found in the caves of the mountains, therefore, it is difficult to obtain. It has been tested as it is common in Oman (already mentioned in the Study Design section. However, this study showed that the application of it as a tissue fixative to replace formalin is encouraging.**

 Also, the preferred pH for tissue fixation should be between 6-8; while at the present research authors used pH of the prepared fixatives "The pH for these fixatives were 7.20, 7.25, 7.27, 3.62, 3.56, 5.15, 3.95, 5.0, and 5.85, respectively." so, I think pH monitoring should be considered.

**Response**

**We measured the different pH for the evaluated nine groups of fixatives, just to show the readers that the tested honey fixatives have a similar pH to that of the** **gold standard fixatives.**

 Figure 1: needed to be at higher magnification to be show the tissue details clearly.

**Response**

**As suggested, a new Figure 1 with a higher magnification has been added.**

 Figure 4: negative immunohistochemical image is needed; also testing another immunohistochemical marker should be applied as vimentin reaction is not so obvious at this figure.

**Response**

**We can add a negative staining picture for vimentin but this looks different from other figures as we present a representative picture for each fixative group and we do not have a negative group in this study. We have chosen vimentin as an immunohistochemical marker for evaluation. In routine immunohistochemistry, this marker is used as an internal control for detecting cytoplasmic staining.**

# Reviewer report: Reviewer response for version 1

**DOI:** 10.5256/f1000research.134611.r174633

**Published:** 2023-07-12

**Authors:** Marie-Odile Benoit-Biancamano

**Affiliations:** 1 Universite de Montreal, Montreal, Québec, Canada

This is an interesting study, but the authors do not explain how it is different from previous studies conducted to test honey as a tissue fixative agent.

 In the abstract, I do not understand what the authors meant by “neutral honey, not natural or artificial honey”. What is neutral honey? And what is it if neither natural nor artificial?

 The authors should be more specific in the abstract regarding the origin of the honey, since the honeys that they used are not commonly known in Eastern countries. Date honey does not come from bees, which should be specified, while Sumer honey is probably near impossible to buy in most countries.

 I do not think that the abstract adequately reflects the results of the study. The authors claim that staining was adequate for all tested conditions, but the detailed results rather showed mitigated to poor results especially regarding the cytoplasmic staining and cell morphology. These are important characteristics for pathologists, as changes are significant on some of the pictures shown and would likely hamper proper diagnosis.

 Page 3 Paragraph 2 Line 3: When describing honey contents here, it is rather confusing if the authors mean honey from bees or honey from fruits.

 Figure 4F was not taken at the same magnification as the others.

 Although this study provides very interesting information on the potential use of honey, the availability of the tested honey types should be discussed. Although they might be common in Oman, those are likely difficult to impossible to obtain in other countries. Even date syrup is rather rare in Eastern countries.

 In the discussion, the authors often use the word “honey” as used in other studies, but it is unclear what type of honey was used in those. Was it fruit honey or bee honey? If bee honey, from what type of bees?

 Minor comments

 Page 3 Paragraph 5 Line 1: Sumar or Sumer?

Is the work clearly and accurately presented and does it cite the current literature?

Yes

If applicable, is the statistical analysis and its interpretation appropriate?

Partly

Are all the source data underlying the results available to ensure full reproducibility?

Yes

Is the study design appropriate and is the work technically sound?

Yes

Are the conclusions drawn adequately supported by the results?

Partly

Are sufficient details of methods and analysis provided to allow replication by others?

Yes

Reviewer Expertise:

Veterinary pathology

I confirm that I have read this submission and believe that I have an appropriate level of expertise to confirm that it is of an acceptable scientific standard, however I have significant reservations, as outlined above.

# Response

**Published:** 2023-07-18

**Authors:** Nasar Alwahaibi

**Affiliations:** Biomedical Science, Sultan Qaboos University, Muscat, Oman

**We would like to take this opportunity to express our thanks to the reviewers for the positive feedback and helpful comments.**

**Below are our responses, point-by-point to the queries of the reviewers.**

 This is an interesting study, but the authors do not explain how it is different from previous studies conducted to test honey as a tissue fixative agent.

**Response**

**Thank you, we have already mentioned in the last paragraph of the introduction section that “Tissues fixed in honey at low pH are less rigid after fixation and have homogenized connective tissue, a breach in the continuity of sections, folding of the tissue sections, and growth of molds over some time. ^1,3,8,9^ The present study was designed to find a safe substitute fixative for formalin without compromising the quality staining criteria of the tissue sections. The hypothesis was that neutral honey fixative or other experimented honey fixed rat tissues better or similar to that of 10% NBF.”**

 In the abstract, I do not understand what the authors meant by “neutral honey, not natural or artificial honey”. What is neutral honey? And what is it if neither natural nor artificial?

**Response**

**In the introduction section, we have defined Neutral honey is a honey where the pH is around seven whereas natural honey usually has a low pH. However, we have added this information in the abstract section as suggested by the reviewer.**

 The authors should be more specific in the abstract regarding the origin of the honey, since the honeys that they used are not commonly known in Eastern countries. Date honey does not come from bees, which should be specified, while Sumer honey is probably near impossible to buy in most countries.

**Response**

**As suggested by the reviewer, we have added the origin type for each honey.**

 I do not think that the abstract adequately reflects the results of the study. The authors claim that staining was adequate for all tested conditions, but the detailed results rather showed mitigated to poor results especially regarding the cytoplasmic staining and cell morphology. These are important characteristics for pathologists, as changes are significant on some of the pictures shown and would likely hamper proper diagnosis.

**Response**

**We meant by the statement “H&E showed adequate staining in all groups compared to 10% NBF” is the nuclear staining. We have corrected this sentence. In addition, we mentioned clearly in the conclusion of the abstract section that further experiments on larger specimens should be conducted.**

 Page 3 Paragraph 2 Line 3: When describing honey contents here, it is rather confusing if the authors mean honey from bees or honey from fruits.

**Response**

**Sorry for the confusion, it is a honey bee and has been corrected.**

 Figure 4F was not taken at the same magnification as the others.

**Response**

**Yes, you are right, it is x20 magnification. A new figure (4f) has been added.**

 Although this study provides very interesting information on the potential use of honey, the availability of the tested honey types should be discussed. Although they might be common in Oman, those are likely difficult to impossible to obtain in other countries. Even date syrup is rather rare in Eastern countries.

**Response**

**As suggested, the following sentence has been added in the discussion section: This study presents two types of honey:** **Sumer (produced by red dwarf bees) and Date honey (produced from dates). These two types of honey are common in Oman. Sumer honey is also available in Saudi Arabia, United Arab Emirates, India, Iran, Iraq, Jordan, Yemen,** **Thailand,** **Cambodia, Vietnam, China,** **and Sudan. Date honey is found in any country that produces dates such as Libya, Algeria,** **Iran, Iraq, Jordan, Yemen,** **Saudi Arabia, and United Arab Emirates. Other countries might have different types of honey.**

 In the discussion, the authors often use the word “honey” as used in other studies, but it is unclear what type of honey was used in those. Was it fruit honey or bee honey? If bee honey, from what type of bees?

**Response**

**We have reviewed each journal cited in the discussion section to find out the type of honey used, and if honey bee used, what type of bees? however, the authors do not specify the types of honey bee, which is the most commonly used honey. Some write pure honey and others pine honey with no more details. We have added this information in the discussion section.**

 Minor comments

 Page 3 Paragraph 5 Line 1: Sumar or Sumer?

**Response**

**It has been corrected; it is Sumer.**
